# Thermomechanical Properties of Polylactic Acid-Graphene Composites: A State-of-the-Art Review for Biomedical Applications

**DOI:** 10.3390/ma10070748

**Published:** 2017-07-04

**Authors:** Ilker S. Bayer

**Affiliations:** Smart Materials, Istituto Italiano di Tecnologia, 16163 Genoa, Italy; ilker.bayer@iit.it; Tel.: +39-380-387-6699

**Keywords:** polylactic acid, polylactide, graphene, PLA, polylactic acid-graphene composite

## Abstract

Due to its biodegradable and bioabsorbable characteristics polylactic acid (PLA) has attracted considerable attention for numerous biomedical applications. Moreover, a number of tissue engineering problems for function restoration of impaired tissues have been addressed by using PLA and its copolymers due to their biocompatibility and distinctive mechanical properties. Recent studies on various stereocomplex formation between enantiomeric PLA, poly(l-lactide) (PLLA) and poly(d-lactide) (PDLA) indicated that stereocomplexation enhances the mechanical properties as well as the thermal- and hydrolysis-resistance of PLA polymers. On the other hand, biomedical application of graphene is a relatively new front with significant potential. Many recent reports have indicated that understanding of graphene-cell (or tissue, organ) interactions; particularly the cellular uptake mechanisms are still challenging. Therefore, use of graphene or graphene oxide properly embedded in suitable PLA matrices can positively impact and accelerate the growth, differentiation, and proliferation of stem cells, conceivably minimizing concerns over cytotoxicity of graphene. As such, PLA-graphene composites hold great promise in tissue engineering, regenerative medicine, and in other biomedical fields. However, since PLA is classified as a hard bio-polyester prone to hydrolysis, understanding and engineering of thermo-mechanical properties of PLA-graphene composites are very crucial for such cutting-edge applications. Hence, this review aims to present an overview of current advances in the preparation and applications of PLA-graphene composites and their properties with focus on various biomedical uses such as scaffolds, drug delivery, cancer therapy, and biological imaging, together with a brief discussion on the challenges and perspectives for future research in this field.

## 1. Introduction

**Figure 1:**
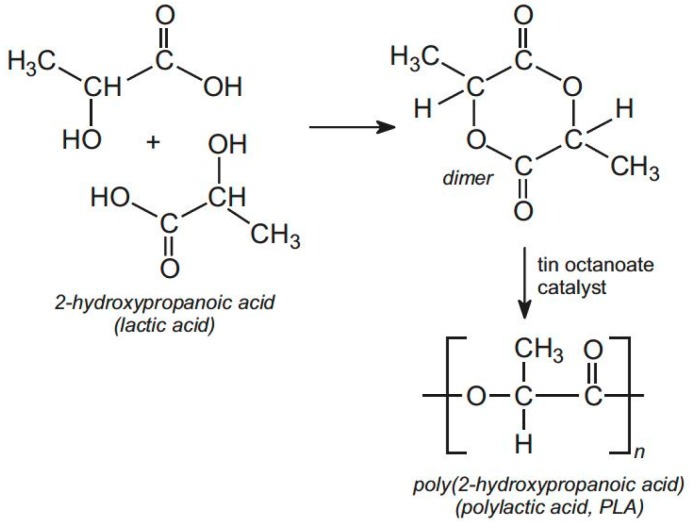
Polymerization of lactic acid to produce polylactic acid (PLA). Lactic acid cannot be polymerized directly as the water produced prevents the polymerization process. The acid is therefore first dimerized by heating and afterwards dimer undergoes ring opening polymerization using tin octanoate as a catalyst. This method avoids the formation of water during polymerization [7].

Polylactic acid (PLA) polymers are synthesized and obtained from renewable agricultural resources by polymerization of lactide, which is the cyclic di-ester of lactic acid [1]. For all practical purposes, lactic acid can be considered as the monomer for PLA [2]. Recent progress in the capability to manufacture the monomer in a cost effective way from renewable feedstocks has placed PLA polymers at the forefront of emerging biodegradable soft materials science. Since lactic acid is a chiral molecule existing in l and d isomers, “polylactic acid” refers to a family of polymers such as poly-l-lactic acid (PLLA), poly-d-lactic acid (PDLA), and poly-d,l-lactic acid (PDLLA). The l-isomer is a biological metabolite and constitutes the main fraction of PLA derived from renewable sources since the majority of lactic acid from biological sources exists in this form [3]. Depending on the composition of the optically active l- and d, l-enantiomers, PLA can crystallize in three forms (α, β, and γ). Thorough understanding of the intrinsic structural, thermal, and physicochemical properties of PLA polymers along with the knowledge of how such properties can be tuned and manipulated with nanomaterials have fueled remarkable biotechnological interest in PLA nanocomposites [4,5,6]. The extensive review by Södergårt and Stold [7] presents different polymerization schemes associated with lactic acid chemistry. A typical metal catalyst driven polymerization mechanism of lactic acid is schematically illustrated in Figure 1. Dimerization of lactic acid needs a priori removal of water in the presence of acidic catalysts. The formation of dimer with high yield and selectivity requires the use of special catalysts which are primarily weakly basic. The use of tin and zinc oxides and organo-stannates and -titanates for this purpose have been reported [8]. Although progress and recent advances in synthesis mechanisms, biocompatibility and biodegradation dynamics of PLA polymers are beyond the scope of this review, a number of excellent review articles are referred to for further reading on these aspects [9,10].

PLA polymers have already been demonstrated as surgical implant materials [11,12,13], drug delivery systems [14,15], guided tissue and bone regeneration platforms, and also as porous scaffolds for the growth of neo-tissue [16,17]. Use of PLA in biomedical applications is not based only on its biodegradability but also on its thermo-mechanical properties including its suitability for shape memory effects [18]. Hence, various medical devices have been prepared from different PLA types including degradable sutures, drug releasing microscale particles, nanoparticles, and porous scaffolds for cellular applications [19]. Copolymers of PLA are also very attractive biodegradable materials for medicine [20,21]. For instance, biodegradation of poly(lactic-*co*-glycolic acid) (PLGA) occurs by hydrolysis leading to metabolite monomers, lactic acid, and glycolic acid. Because these two monomers are endogenous and easily metabolized by the body via the Krebs cycle, a minimal systemic toxicity is associated with the use of PLGA as biomedical material and drug releasing vehicle [22].

According to Rasal et al. [23], PLA polymers have a number of limitations. These can be summarized in three groups: mechanical limitations, biodegradation rates and byproducts, chemical reactivity and hydrolytic degradation. PLA is very brittle polymer. Although its tensile strength and elastic modulus are comparable to poly(ethylene terephthalate) its poor toughness limits its use in applications where plastic deformation at higher stress levels is required such as implant screws and fracture fixation plates. For instance, when poly-l-lactic acid interference screws were used for graft fixation during anterior cruciate ligament (ACL) reconstruction, the devices were reported to be mechanically weaker than their metallic counterparts and often fractured during implantation [23]. Secondly, its biodegradation rate is considered to be quite slow (3 years or more), which depends on its crystallinity and molecular weight. This causes concerns over secondary surgical procedures for implants made with PLA [24]. However, recent studies indicate that laser treatment of PLA polymers can accelerate their biodegradation rates [25]. Moreover, acidic byproducts during its degradation in vivo have been causing concerns in surgical applications [26,27]. Thirdly, an important limitation is its water sensitivity, which is reflected through interaction with moisture, resulting in hydrolytic destruction or hydrolysis [28,29]. Nonetheless, aforementioned limitations can be addressed by using PLA copolymers, adding bio-based plasticizers, designing PLA nanocomposites, grafting other polymers [30] and blending PLA polymers with faster biodegrading polymers or enzymes to name but a few [31]. Stereo-complexes of PLLA and PDLA deserve special attention due to the fact that PLA stereo-complexes have higher melting temperature (or heat resistance), mechanical performance, and hydrolysis-resistance compared to those of neat PLLA and PDLA [32].

Graphene, on the other hand, has been at the forefront in biotechnological applications [33,34,35,36]. Graphene is a free-standing 2D crystal with one-atom thickness. It is in fact an allotrope of carbon comprising layers of six-atom rings in a honeycombed network and can be theoretically viewed as a true planar aromatic macromolecule [37,38]. Graphene exhibits remarkable properties [39], such as unusual electronic flexibility as well as high planar surface area (~2630 m^2^/g), outstanding mechanical strength (Young’s modulus, ~1100 GPa) and unparalleled thermal conductivity (~5000 W/m/K). As such, it is a highly popular nanoscale additive for reinforced biopolymers [40]. Biosafety of graphene and graphene derivatives is attracting more and more attention as we need to learn the fate of graphene and its derivatives in vivo once it invasively enters into a biological system [39,40,41]. It has now been acknowledged that the biological effect of graphene is complex and largely affected by the exposure dose and methods as well as the chemical functionalization, lateral size, thickness, and surface properties [42,43].

**Figure 2:**
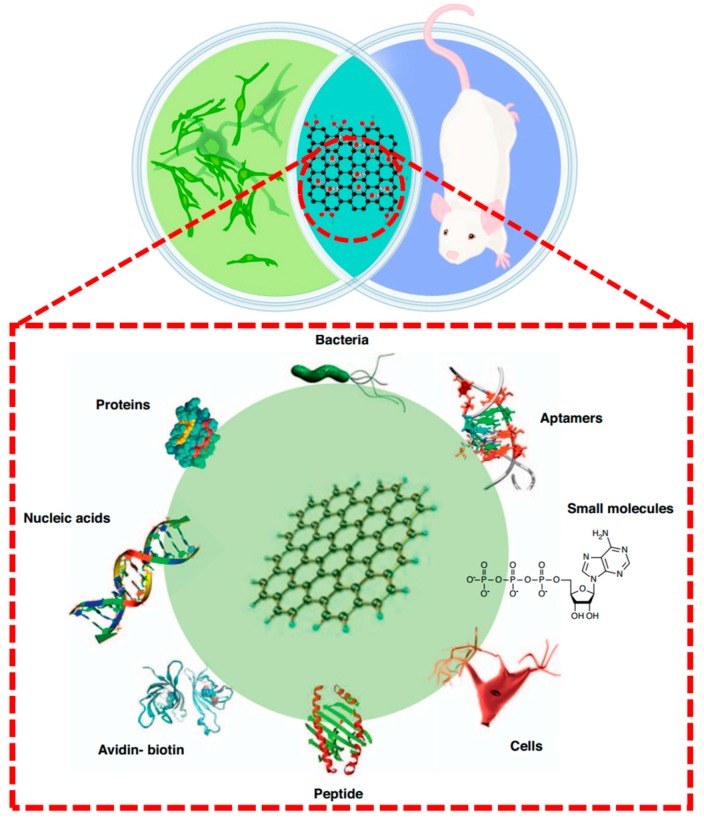
Although graphene oxide has been demonstrated to be cytocompatible in vitro, its compatibility in vivo in tissue sites relevant for biomedical device application is yet to be fully understood. Promising results may be achieved by proper functionalization. Graphene and its derivatives have been reported to be functionalized with avidin–biotin, peptides, nucleic acid, proteins, aptamers, small molecules, bacteria, and cells through physical adsorption or chemical conjugation. Reproduced from [37,45].

Among graphene derivatives, graphene oxide (GO) has been widely explored for in vitro and in vivo drug delivery and imaging, taking advantage of its high solubility and stability in physiological solutions, low cost and scalable production, and facile biological/chemical functionalization [41,42,43,44]. Several studies reported good biocompatibility [45,46]. Nevertheless, granuloma formation, inflammation, and thrombus formation in mice have also been observed after GO exposure [47,48]. Both graphene and graphene oxide can be adapted to function as smart biomedical devices as long as their surfaces are properly functionalized. Figure 2, for instance, schematically illustrates several potential functionalization approaches to render graphene and graphene oxide cytocompatible both in vitro and in vivo. Among them proteins, small biological molecules, DNA and peptides are very popular and promising. Note that ideally graphene is a single atomic layer planar material but almost all the polymer nanocomposites made with graphene utilize flakes that are made up of many layers of “real grapheme” bundled stacked up form. They are generally referred to as graphene platelets, graphene nanoplatelets, graphene nanosheets, etc. and are denoted by GPs, GnPs, xGnPs, etc.

As such, thermo-mechanical properties of PLA polymers are directly related to their biomedical performance when interfaced with biological systems, since these properties can be used to optimize important design criteria such as modulus, strength, morphology, crystallinity, biocompatibility, and, in turn, these properties can affect cell response, tissue regeneration, and in vivo degradation. Moreover, recent research indicates that thermal effects on the crystallinity of not only PLA polymers but also other bio-polyesters is very critical for cell biocompatibility and drug release dynamics [49]. Since, nowadays graphene and its derivatives have been shown to influence thermo-mechanical properties of many semi-crystalline plastics including biopolymers, carefully designed and engineered nanocomposites of PLAs with graphene and its derivatives can be used to remediate certain structural, thermal, and degradation related drawbacks of PLA polymers making them more attractive for biomedical applications. Moreover, research efforts on biopolymer-graphene/GO-based scaffold materials for cell culture is an ever expanding field that deserves special attention since graphene and GO are able to accelerate growth, differentiation, and proliferation of stem cells, and therefore hold great promise in tissue engineering, regenerative medicine, and other biomedical fields.

## 2. Thermal Effects on the Crystallinity of PLA Polymers

One of the most important performance comparison criteria between biodegradable polymers and conventional oil-based polymers is thermal stability along with moisture retention issues. Although mechanical properties of PLA polymers match those of poly(ethylene terephthalate) (PET) polyesters, for instance, their thermal stability is not as high as PET polymers [50]. Thermal stabilization of PLA polymers are closely related to their crystallization behavior. Moreover, PLA’s wider application has been limited by its relatively slow crystallization rate from the practical application viewpoint. However, new recent research shows that crystallization of PLA polymers can be tuned by preparing homo-composites (based purely on PLA polymers with different structures) [51].

**Figure 3:**
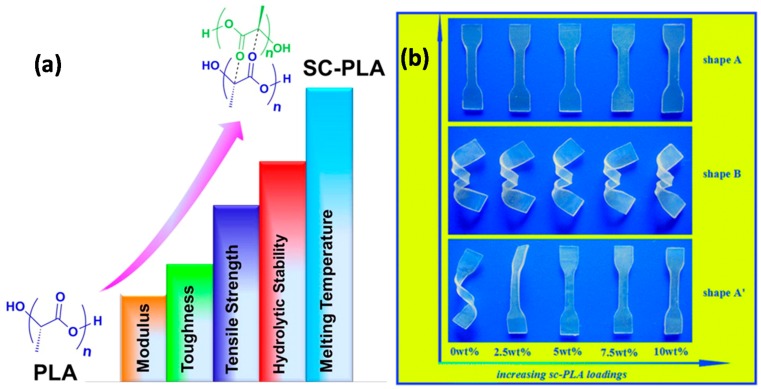
(**a**) Schematic representation of thermal and mechanical enhancement possibilities of PLA via stereo-complexation of PLA in different polymeric systems, including enantiomeric PLA homopolymers and PLA-based block and graft copolymers, as well as enantiomeric PLA materials; (**b**) Photographs of the shape recovery of neat PDLLA and the blend samples of PDLLA with increasing stereo-complex PLA loadings [52].

A comprehensive review by Tan et al. [52] illustrates that diverse isomeric forms of PLA could provide great opportunities for thermal and mechanical enhancement through stereo-complexation. In their review, recent developments in thermal and mechanical enhancement of PLA via stereo-complexation in different polymeric systems were presented. They include enantiomeric PLA homopolymers, PLA-based block and graft copolymers, as well as enantiomeric PLA materials having unique architectures such as cyclic, star, dendritic and comb-shaped morphologies. Insightful discussions on the influence of crystal structure and intermolecular interactions between PLLA and PDLA in different polymeric systems on performance enhancement of the resultant materials were discussed. The “enhanced” PLA polymers with diverse functions oriented toward engineering materials and their biomedical significance in various areas were also covered in their review. Figure 3a schematically shows that almost all mechanical, thermal, and hydrolytic properties of PLA polymer can be improved by stereo-complexation. As an example, incorporating stereo-complex PLA in PDLLA polymer, thus forming homocomposites, grants unprecedented shape recovery ability to the neat PDLLA resin (see Figure 3b).

**Figure 4:**
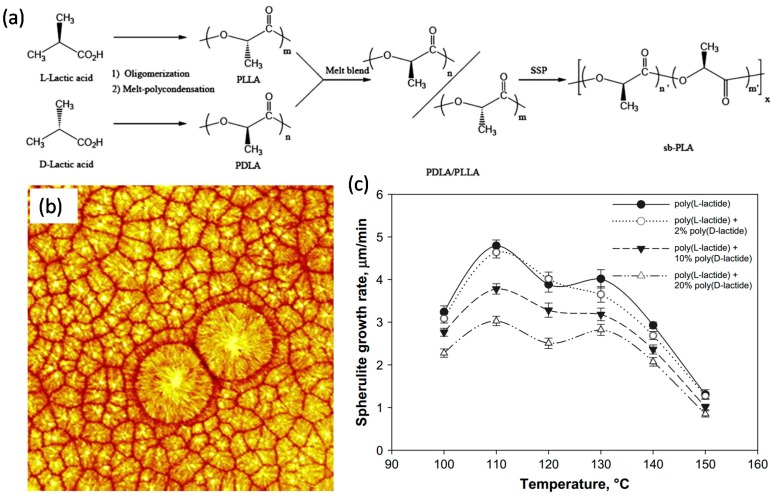
(**a**) Synthesis of sb-PLAs with high-molecular weight by solid-state polycondensation (SSP) of the melt blend (PLLA/PDLA); (**b**) Typical crystalline structure of PDLA annealed at 110 °C. The square image size is 100 μm; (**c**) Isothermal spherulite radius growth rates for PLLA and its blends with PDLA. Reproduced from [53,54].

Hirata and Kimura [53] conducted a careful experimental study on thermomechanical properties of stereoblock PLAs with different PLLA/PDLA block compositions. They synthesized stereoblock PLAs (sb-PLAs) having abundant enantiomeric compositions of PLLA by solid-state polycondensation of the melt blends of medium molecular weight prepolymers. Their stereoblock PLA chemical synthesis method is shown in Figure 4a. Resulting sb-PLAs were cast into polymer films by solution deposition. Dynamic mechanical analysis measurements signified the retention of storage modulus above melting point T_m_ of homo-chiral crystals (160 °C). They argued that PLLA-rich sb-PLAs can be used as more robust and high-performance resins compared to PLA; minimizing the use of d-lactic acid which is more expensive than l-lactic acid.

Yuryev et al. [54] studied surface crystallinity on films of poly(l-lactide), poly(l/d-lactide) and their blends with poly(d-lactide). The isothermal spherulitic crystal growth rate and its dependence on temperature were measured using tapping mode atomic force microscopy and ex-situ isothermal crystallization. Further, they also investigated the crystallization kinetics of blends of poly(l-lactide) and poly(l/d-lactide) with poly(d-lactide). The typical crystalline structure of PDLA polymer annealed at 100 °C can be seen in the atomic force microscope image in Figure 4b. Resultant crystalline domain growth rate as a function of PDLA in PLLA is shown in Figure 4c. At any given temperature below 150 °C, adding more PDLA (as percentage in stereoblock polymer) results in lowering of the spherulite growth rate compared to pure PLLA. These polymers are still rich in PLLA (not more than 20% PDLA; see Figure 4c) but with much better crystalline structure.

Perego et al. [55] analyzed several samples of PLA polymers with different molecular weights and tacticity to investigate the effect of molecular weight and crystallinity on mechanical properties. Injection molded PLA specimens were annealed to promote their crystallization. From the characterization data, PLLA showed more interesting mechanical properties than PDLA, and its behavior significantly improved with crystallization. This is attributed to the stereo-regular nature of the poly(l-lactic acid) chain. Thermally annealed specimens retained higher values of tensional and flexural modulus of elasticity, impact strength, and heat resistance. They also showed that while PDLLA and amorphous PLLA presented a heat resistance very near to their glass transition temperature (T_g_), annealed PLLA demonstrated a better behavior in the sense that it was more stable at temperatures of 50 °C or higher, due to its crystallinity induced stability.

**Figure 5:**
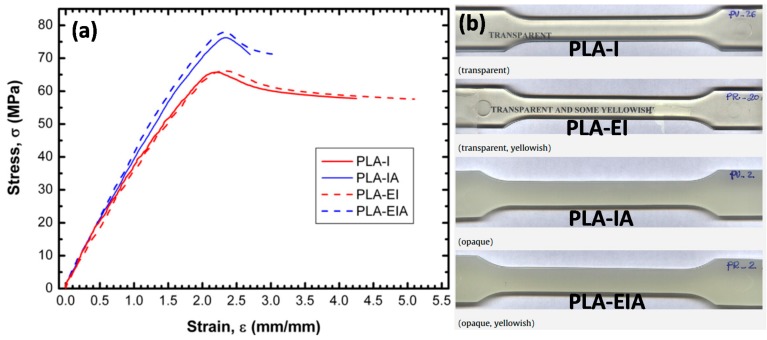
(**a**) Representative stress vs. strain curves from tensile testing for processed PLA polymers. Nomenclature; PLA-I: injection molded; PLA-EI: extruded and injection molded; PLA-IA: injection molded and annealed PLA-EIA: extruded, injection molded and annealed; (**b**) Physical appearance of the thermo-processed specimens [56].

In a highly cited work, Carrasco et al. [56] processed PLA resin with injection and extrusion/injection as well as annealing. These thermally processed PLAs were studied in order to analyze the variations in chemical structure, thermal degradation, and mechanical properties (see Figure 5). Processing of PLA demonstrated evidence for a decrease in molecular weight due to chain scission. They found that mechanical processing led to quasi disappearance of crystal structure whereas it was recovered after post-annealing. After careful analysis of Fourier transform and Nuclear Magnetic Resonance spectroscopy chemical shifts and peak areas, they affirmed that the chemical composition of PLA did not change after processing, but the proportion of methyl groups increased, thus indicating the presence of a different molecular environment. In fact, they showed that annealed specimens possessed higher values of tensional and flexural modulus of elasticity, Izod impact strength, and heat resistance. After annealing, samples showed an increase in Young modulus (5–11%) and in yield strength (15–18%), which was explained by the higher degree of crystallinity of annealed materials, with a subsequent decrease in chain mobility. Extruded/injected polymers showed a significant increase in elongation at break (32–35% more), compared to injected materials. This was attributed to a higher number of chains due to chain scissions in reprocessed materials (see Figure 5).

As can be seen from this section, crystallization of PLA polymers is a very complex thermal process. Many factors such as post annealing, aging, stereocomplexation, cooling or heating rates, plasticizers not only play a significant role in the crystallization temperature but also in the shape, size, type, and density of the crystals that would nucleate and form, eventually determining the final thermomechanical properties of the polymer.

## 3. Effect of PLA Polymers Crystallinity on Cells

The unique crystalline properties of PLA polymers render them attractive platforms for controlling cellular behavior. In fact, homo-composites of PLA polymers with different crystallinity or engineering PLA polymers with crystallinity gradients have been inspired by the occurrence of natural tissues presenting continuous variations of properties along one direction, such as ligament-to-bone, tendon-to-bone, and dentin-to-enamel interfaces [57,58]. The pioneering work by Simon Jr. et al. [58] demonstrated a rapid method for screening cell proliferation on PLLA/PDLLA blends. Strip-shaped films containing a gradient in polymer composition were prepared and characterized. Cells were cultured on the films and adhesion and proliferation were assessed using high-throughput, automated microscopy. More specifically, they combined automated fluorescence microscopy with a combinatorial approach for creating polymer blend gradients to yield a rapid screening method for characterizing cell proliferation on polymer blends.

**Figure 6:**
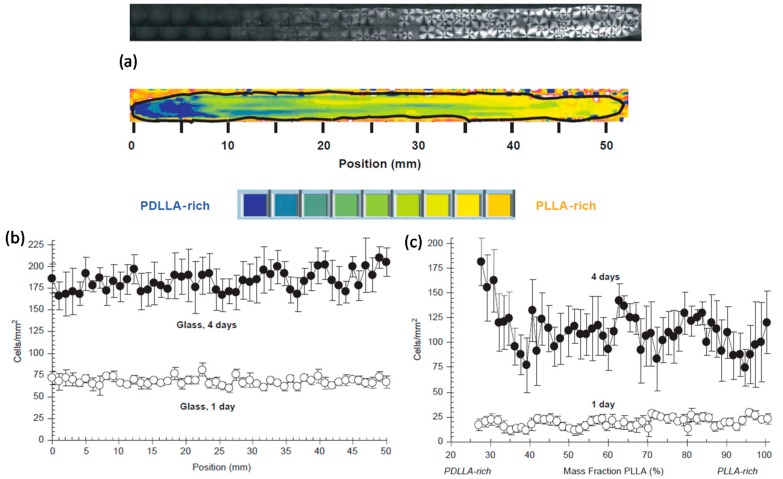
(**a**) Top: An image of the birefringence from a PLLA-PDLLA gradient is shown. The higher crystallinity of the PLLA-rich end of the gradient causes it to be more birefringent than the PDLLA-rich end. The gradient shown is 52 mm long. Bottom: FTIR-reflectance-transmission microspectroscopy (RTM) map of a PLLA-PDLLA composition gradient. The strip film has been outlined with a black line. Blue corresponds to PDLLA rich regions and orange to PLLA-rich regions (see color bar below map); (**b**) Cell adhesion and proliferation on the PLLA-PDLLA gradients and on control glass slides. Adhesion data for 1 day and for proliferation data for 4 days are plotted; (**c**) Same data as b on the PLLA-PDLLA gradient substrate [58].

Strip-shaped blend films were annealed to allow PLLA to crystallize as shown in Figure 6a. Fourier transform infrared (FTIR) micro-spectroscopy was used to determine the composition in the gradients and atomic force microscopy was used to characterize surface topography (also shown in Figure 6a). Osteoblasts were cultured on the gradients and proliferation was assessed by automated counting of cells using fluorescence microscopy as shown in Figure 6c; whereas Figure 6b demonstrates their control experiments on glass slides. Surface roughness varied with composition, PDLLA-rich regions were smooth whereas PLLA-rich regions were rough. The enhanced cell proliferation on PDLLA-rich regions of the gradients correlated with this smooth topography but the authors could not exactly conclude whether this was a result of blend composition or surface roughness since both parameters had been varied across the gradients. They argued that for the case of PLLA-PDLLA blends, smooth PDLLA-rich blends could be best suited for supporting cell proliferation. Adhesion was similar in all regions of the gradients after one day of observation but after five days proliferation was enhanced on the PDLLA-rich ends of the gradients.

**Figure 7:**
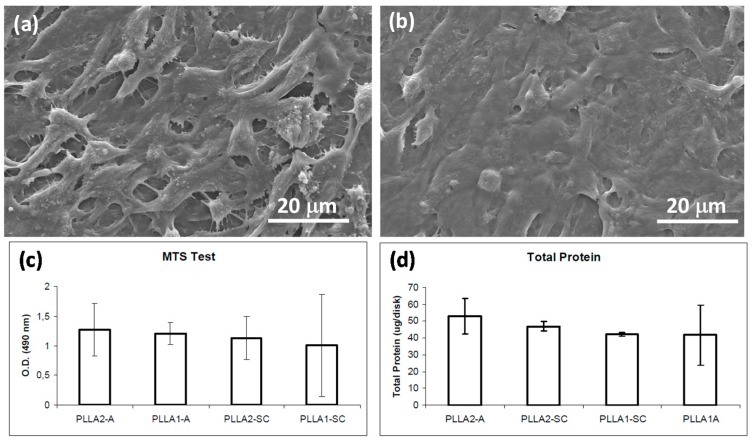
SEM micrographs of human osteoblasts like cells after two weeks in culture (**a**) amorphous PLLA1; (**b**) semi-crystalline PLLA1; (**c**) Cell viability measured from the optical density (O.D.) at 490 nm; and (**d**) Cell proliferation assays on SaOS-2 cells grown on PLLA disks, (PLLA1—low molecular weight; PLLA2—high molecular weight; A—amorphous; SC—Semi-crystalline) [59].

Sladago et al. [59] reported a study to evaluate how crystallinity and molecular weight of PLLA influenced the patterns of cell adhesion, proliferation, and cell morphology parameters. Four conditions were tested: low molecular weight amorphous and semi-crystalline hot-pressed PLLA disks (PLLA1-A and PLLA1-SC), and high molecular weight amorphous and semi-crystalline hot-pressed PLLA disks (PLLA2-A and PLLA2-SC). For the cell culture studies they chose a human osteosarcoma cell line (SaOS-2). Disks were immersed in a cell suspension containing 5 × 10^4^ cells/mL and kept in culture for periods up to two weeks. They concluded that cell viability was not affected by the different tested conditions. However, cell proliferation was increased in the high molecular weight amorphous samples and cells seemed to have higher adhesion patterns on semi-crystalline samples. They attributed these observations to different rates of integrin interaction with the substrate leading to different patterns of focal adhesion point formation. Figure 7 shows a summary of their results including scanning electron micrographs of the cells on the PLLA surfaces (Figure 7a,b).

**Figure 8:**
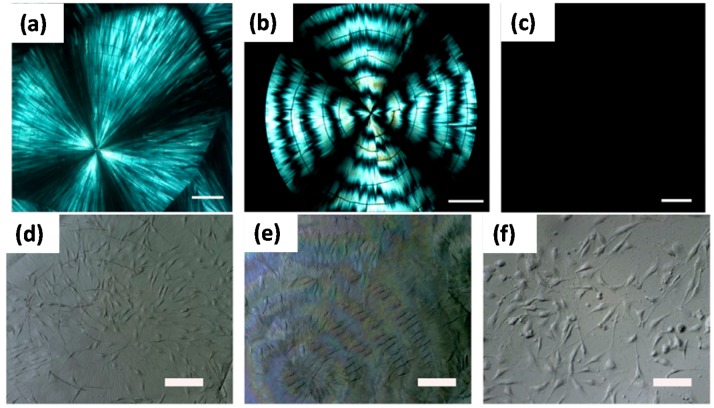
Polarized optical images of PLLA films crystallized at various crystallization temperature (T_c_) values. (**a**) T_c_ = 150 °C; normal spherulite surface (**b**) T_c_ = 145 °C; ring-banded spherulite (**c**) melt-quenched sample; amorphous surface. The scale bar represents 100 μm. Stereo microscope images of MC3T3-E1 cells cultured on PLLA surfaces; (**d**) normal spherulite surface; (**e**) ring-banded spherulite surface; (**f**) amorphous surface. The scale bar represents 100 μm [60].

Li et al. [60] used PLLA films having normal spherulites (Figure 8a), banded spherulites (Figure 8b), and amorphous surfaces (Figure 8c) as model substrates to conduct a systematic assessment of the role for polymer crystallization induced surface morphologies on cell growth and contact guidance. Figure 8 shows that the orientation of cells was along the crystal spherulite radial direction on normal and banded spherulite surfaces. However, on amorphous surfaces, cells spread out in a random fashion because amorphous samples do not contain any special patterned structure (Figure 8d,f). The cell shape, spreading, and orientation were investigated by fluorescent staining techniques in order to find the possible correlation between the cells behavior and the crystallization morphology of PLLA.

They used microscopy and image analysis to ascertain that the MC3T3-E1 cells spread out in a random fashion on the amorphous PLA. At 24 h post-seeding, MC3T3-E1 cells on both types of spherulite surfaces were elongated and aligned along the spherulite radius direction. For the banded spherulite surface with radial stripes and coupling annular grooves, the cell orientation and cell nuclear localization were related to the groove structure. With increasing time, this orientation preference was weaker. They further demonstrated that the patterning of the polymer crystallization structure provides important indicators for guiding cells to exhibit characteristic orientation and morphology especially in the early stages of regeneration.

**Figure 9:**
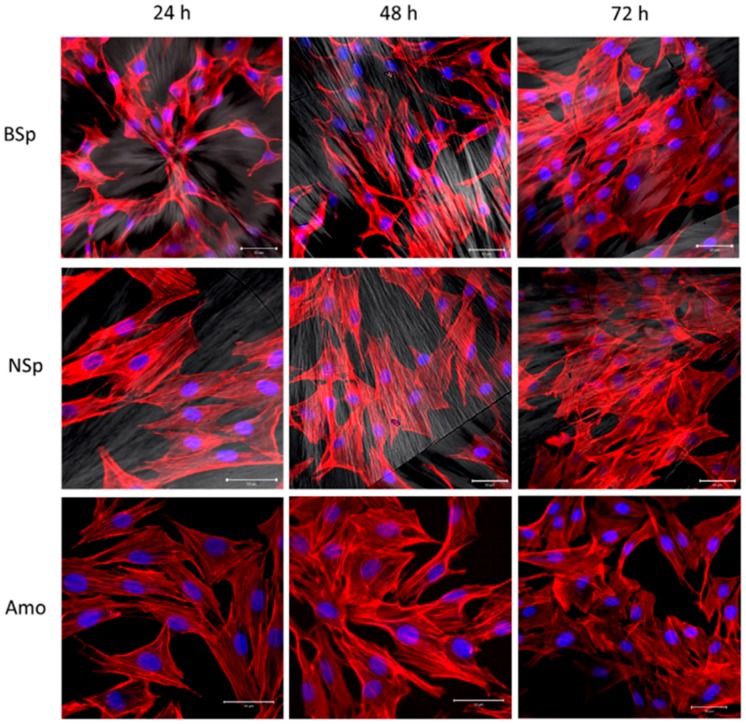
Morphology of MC3T3-E1 cells cultured on PLLA surfaces. Cells were stained with Alexa Fluor 568 (red) and DAPI (4′,[6]-diamidino-2-phenylindole; blue). The scale bar represents 50 μm. Crystallization temperatures for normal spherulite samples (NSp) and ring-banded spherulite samples (BSp) were 150 and 143–147 °C, respectively. For preparing amorphous PLLA films (Amo), the molten state samples were quenched into ice water [60].

Figure 9 shows the three PLLA substratum samples for three time points. On non-patterned amorphous PLLA surface, MC3T3-E1 cells spread extensively with overall cell morphology showing no preferred direction. The actin arranged mainly around the periphery of the cells. On normal spherulite and banded spherulite films, MC3T3-E1 cells exhibited an elongated morphology. The spread direction of cells cytoskeleton was basically consistent with the radial direction of the spherulite. In particular, for the banded spherulite surface having radial stripes and coupling annular grooves, there were two competing factors to guide the cells’ behavior. The radical guidance preference decreased with increasing valley width. On the other hand, the percentage of cell nucleus located in the valley of the banded spherulitic structure increased with increasing valley width.

Using a novel high-throughput method for creating gradients in polymer crystallinity, Washburn et al. [61] demonstrated that cells (MC3T3-E1 osteoblastic cells) were exquisitely sensitive to variations in nanometer-scale PLLA surface topography. They found that cells were sensitive to topographic features of the order of 5 nm and that the observed inhibition of proliferation did not seem to be mediated through changes in adherent proteins but rather seemed to be directly related to changes in substrate roughness. Their results suggested that cells were much more sensitive to topography than previously expected, and the inhibition of proliferation could be mediated through other mechanisms. The down-regulation of cell division on crystalline surfaces compared with the up-regulation on phase-separated polymer blends indicated that the details of topographic organization were capable of exerting both positive and negative influences on proliferation.

**Figure 10:**
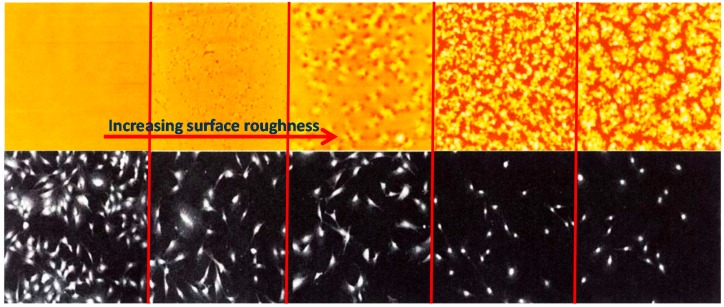
Montage of representative images of PLLA morphology from AFM data (top panels, field of view in each image is 20 μm), and corresponding cell count from fluorescent microscopy (bottom panels, field of view in each image is 1500 μm) [61].

Figure 10 indeed shows their results as increasing roughness causing significant decline in the cell proliferation (from left to right). They also conducted fluorescence microscopy imaging of anti-paxillin-stained cells on amorphous PLLA, PLLA with intermediate crystallinity, and fully crystalline PLLA and reported that no significant variation in the number, position or shape of the adhesion plaques was observed across the library [61].

## 4. Effect of Graphene on the Crystallization of PLA Polymers

PLA polymer-graphene nanocomposites have been increasingly gaining in popularity due to attractive features offered by both materials [62,63]. Norazlina and Kamal [64] reviewed advances in the fabrication of PLA/graphene nanocomposite in detail including requirements for chemical modification of graphene for proper dispersion. However, their review did not present any potential biomedical attributes of PLA/graphene nanocomposites. Fabrication of PLA-graphene nanocomposites were described and classified according to in situ polymerization, solution, and melt blending; and the properties of these nanocomposites were reviewed. The main conclusion was that unless graphene could be chemically modified to enable good dispersion within the PLA matrix, no enhancements in mechanical properties would be possible. On the other hand, there is intense biomedical interest in PLA-graphene nanocomposites based on electrospinning fabrication methods, i.e., nanofiber mats of PLA or PLA copolymers containing graphene or graphene oxide; as they are considered to be good candidates for biomedical scaffolds. Advances in this area will be reviewed after this section, which will cover the effect of graphene on the crystallinity of PLA polymers.

**Figure 11:**
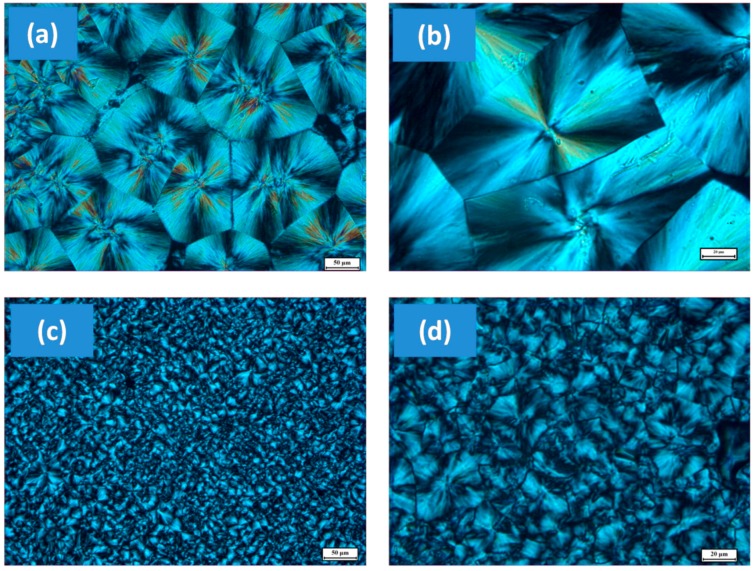
Polarization photomicrographs of the final morphologies of (**a**,**b**) neat PLA and (**c**,**d**) PLAC (PLA-graphene composite) after melt crystallization (120 °C); The scale bars are (**a**,**c**) 50 and (**b**,**d**) 20 μm [65].

Wu et al. [65] conducted a systematic study on the crystallization of PLA in the presence of graphene nano-platelets or nanosheets. Concentration of graphene nanosheets was maintained at 1 wt% with respect to the polymer. They used solution mixing to disperse graphene in the PLA matrix. The presence of graphene nanosheets had a significant influence on both the cold and melt crystallization dynamics of PLA polymer. Upon addition of graphene nanosheets, the overall rate of cold crystallization decreased, and the graphene nanosheets acted merely as inert fillers. For melt crystallization, however, the presence of graphene nanosheets gave rise to a heterogeneous nucleating effect and, as a result, accelerated the overall crystallization process despite the physical hindrance to PLA chain diffusion. Wu et al. [65] further stated that although both PLA and the composite were hard to crystallize in a non-isothermal quenching process, they could be crystallized by isothermal quenching from the molten state, as can be seen in Figure 11. The spherulitic morphology is very clear in the figure for both PLA and the graphene-composite, indicating that the three-dimensional growth of PLA crystals is unchanged by the addition of graphene nanosheets. However, the presence of graphene nanosheets prevents large crystalline domains from forming, resulting in smaller crystalline domains and/or a degraded crystallite structure (Figure 11b). This is indicative of a heterogeneous nucleating effect.

Wang et al. [66] prepared a series of biodegradable PLLA/GO nanocomposites using various graphene oxide (GO) loadings ranging from 0.5 to 2 wt% using DMF as a mutual solvent. They showed that non-isothermal melt crystallization peak temperatures were slightly higher in the GO nanocomposites than in neat PLLA; moreover, the overall isothermal melt crystallization rates were significantly greater in the nanocomposites than in neat PLLA. They postulated that GO may act as a nucleating agent for PLLA. With increasing crystallization temperature, the overall isothermal melt crystallization rates were found to be reduced for both neat PLLA and the PLLA/GO nanocomposites at different GO loadings. More importantly, they showed that both the non-isothermal melt crystallization peak temperature and the overall isothermal melt crystallization rate of PLLA first increased and then decreased with increasing the GO loading from 0.5 to 2 wt% in the PLLA/GO nanocomposites, showing a maximum at a 1 wt% GO loading. Polarized microscope analysis results were similar to the results of Wu et al. [65] in the sense that GO forced formation of smaller spherulitic features.

Manafi et al. [67] prepared nanocomposites based on PLA/graphene nanoplatelets (GnPs) by solution mixing. To improve the dispersion of GnPs in the matrix, functionalization using acid treatment and acylation reaction was performed. Characterization of functionalization reaction and grafting reaction between PLA and functionalized GnPs was verified by spectroscopic analysis and thermogravimetry. Transmission electron microscopy (TEM) results demonstrated that a relatively fine dispersion of GnPs was achieved in the PLA matrix. Non-isothermal tests revealed that the crystallization temperature (T_c_) was increased in samples due to better dispersion and grafting reaction between GnPs and PLA. Isothermal tests also showed that commencement of the crystallization time was affected by the functionalization and then decreased. They also concluded that the growth mechanism of crystallization did not change significantly.

**Figure 12:**
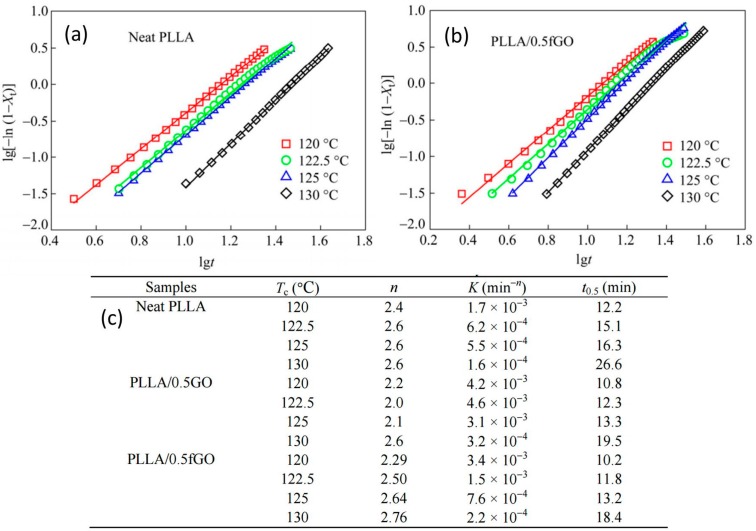
Avrami plots of neat PLLA (**a**), and PLLA/0.5fGO (**b**). Isothermal crystallization kinetics parameters of neat PLLA, PLLA/0.5GO and PLLA/0.5fGO obtained by Avrami fit are tabulated in (**c**) [68].

The effects of GO with polar groups and functionalized graphene oxide (fGO) with non-polar groups on the isothermal crystallization of PLLA were studied by Zhao et al. [68]. Functionalized GO was obtained by grafting octadecylamine and characterized by Fourier transform infrared spectroscopy, X-ray diffraction, and thermogravimetry. The isothermal crystallization kinetics of PLLA/GO and PLLA/fGO nanocomposites was investigated by combining differential scanning calorimetry data and the Avrami equation as shown in Figure 12. Note that the Avrami equation describes how solids transform from one phase (state of matter) to another at constant temperature. It can specifically describe the kinetics of crystallization. The results showed that fGO could improve the PLLA crystallization rate more apparently than GO. By analyzing the morphology obtained from polarized light microscopy, SEM and TEM, it was found that fGO with large layer space dispersed better in PLLA and supplied more nucleation sites than GO. Therefore, for the multilayer graphene, increasing the layer spaces is important to improve its dispersion in polymers, which will cause the modification of crystallization kinetics. According to Figure 12, PLLA/0.5fGO (0.5% fGO dispersion in the polymer matrix) has the best crystallization half-time results at each crystallization temperature (T_c_) studied by Zhao [68].

**Figure 13:**
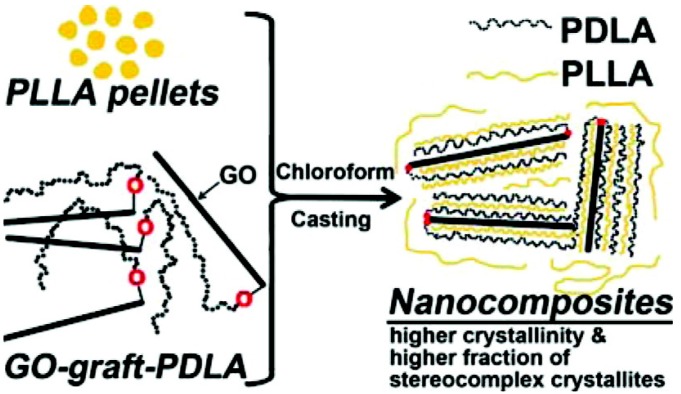
Schematic representation of GO grafting to PDLA and blending in solution for casting stereocomplex homocomposites [69].

Sun and He [69] prepared PLA–graphene oxide (GO) nanocomposites by blending commercial PLLA with PDLA grafted GO (GO-g-PDLA), where GO-g-PDLA was synthesized via ring-opening polymerization using modified GO as the initiator (see Figure 13). Their Fourier transform infrared spectroscopy, differential scanning calorimetry, and X-ray diffraction studies showed that a stereo-complex crystal could be formed between PLLA and GO-g-PDLA. The incorporation of GO nanosheets led to a lower crystallization activation energy of stereo-complex and a higher crystallinity in solution casted samples, mainly due to the heterogeneous nucleating effect of the well-dispersed covalently bonded GO sheets, while in cold crystallized samples, the crystallinity was found to be low owing to exfoliated GO sheets which, they argued, could reduce chain mobility and hinder crystal growth.

**Figure 14:**
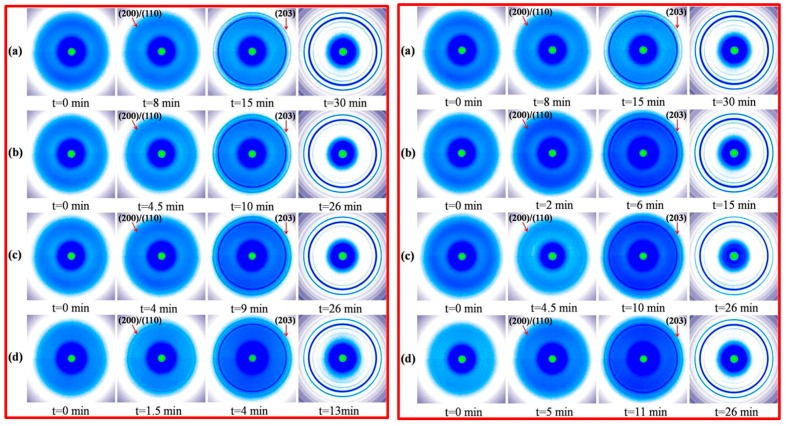
**Left panel**: Selected 2D-WAXD patterns of (**a**) neat PLA; (**b**) PLA-rGOw 0.5; (**c**) PLA-rGOw 1.0; and (**d**) PLA-rGOw 2.0 isothermally crystallized at 135 °C. **Right panel**: selected 2D-WAXD patterns of (**a**) neat PLA; (**b**) PLA-rGOp 0.3; (**c**) PLA-rGOp 0.6; and (**d**) PLA-rGOp 1.0 isothermally crystallized at 135 °C [70].

Liang et al. [70] studied the role of size and structural integrity of thermally reduced graphene oxide (rGO) in PLA crystallization. Reduced graphene oxide nanoplatelets with different architectures were obtained via bath and probe ultrasound processing. Isothermal crystallization kinetics of PLA nanocomposites containing bath (rGOw) and probe ultrasound processed (rGOp) was determined by in situ synchrotron wide-angle X-ray diffraction. The induction period and overall crystallization rate of PLA-rGO nanocomposites were strengthened with diminishing platelet size because of more nucleation sites encouraged by redistribution of functional groups. Figure 14 illustrates the representative 2D-WAXD patterns of PLA-rGOw and PLA-rGOp nanocomposites isothermally crystallized at T_c_ of 135 °C. They observed a diffuse scattering ring in the first pattern (t = 0 min) for all the samples, indicating no crystals could have survived in the melt. With increasing crystallization time, the isotropic lattice planes, i.e., (200)/(110) reflection, can be observed and gradually sharpen until the completion of crystallization, correlating with the formation and growth of the PLA crystals. In contrast to neat PLA, the advent of ultrasonically treated rGO shortens the appearance time of the diffraction peak remarkably (from 30 min to lower values), demonstrating the superb nucleation ability of rGO.

Their main conclusion was that both rGOw and rGOp could serve as heterogeneous nucleation agents for PLA, decreasing the induction period of crystallization and accelerating the overall crystallization rate. Prolonged ultrasound time, however, gradually enhanced the nucleation ability of rGOw but suppressed that of RGOp.

Xu et al. [71] fabricated mechanically strong and thermally stable PLA films by integrating stereocomplex crystals (SCs)-decorated graphene oxide (GO) nanosheets in enantiomeric PLA. In the PLA matrix, GO nanosheets were homogeneously dispersed and fully extended, principally due to the relatively low GO loadings and adequate interactions between GO and PLA. Such morphological features resulted in the construction of interconnected networks of GO nanosheets. Their examination of isothermal and non-isothermal crystallization showed that the selectively accelerated stereocomplex crystallization in the presence of GO rendered a dominant generation of SCs directly proportional to GO loadings. At the same time, the generation of homocrystals (HCs) was suppressed, primarily due to the limited availability of homochiral chains and spatial hindrance caused by the surrounding nanosheets and SCs. More importantly, the decoration of GO with ordered PLA lamellae served as strong and resilient ligaments, which resulted in low gas barrier properties, high strength, high ductility, and improved chemical resistance for GO/PLA composites.

**Figure 15:**
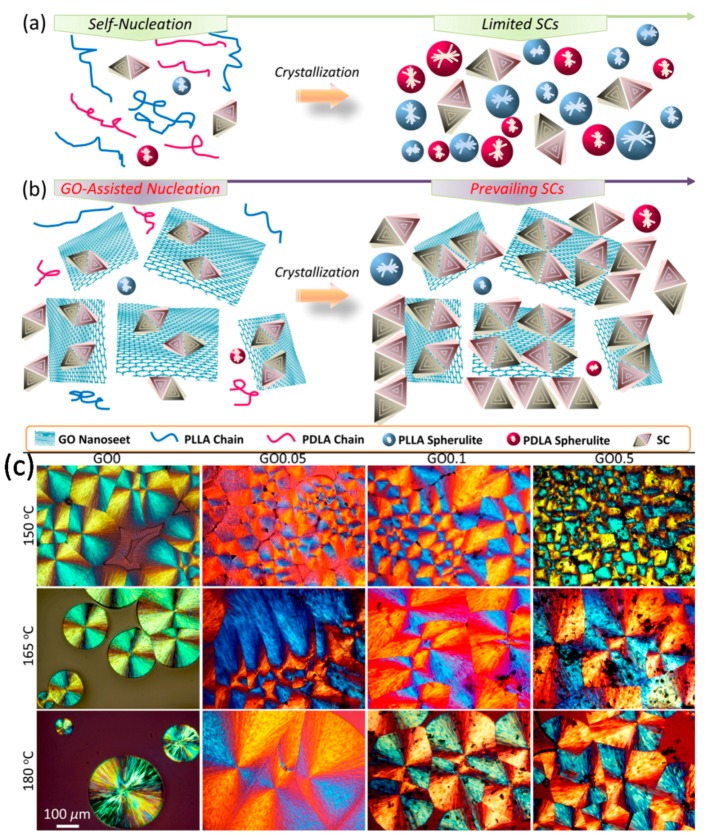
Schematic representation comparing the evolution of homochiral and stereocomplex crystallization in (**a**) GO0 (no graphene oxide) and (**b**) GO reinforced composites. Preferential nucleation of SCs assisted by GO, both on the basal planes and at the edges, leads to the dominating development of SCs and spatially hindered homocrystals, whereas limited SCs are generated during the simultaneous growth of HCs for GO0. (**c**) Polarized optical micrographs showing the spherulitic textures formed in GO/racemic PLA composites after isothermal melt crystallization. The nucleation activity of PLA was evidently enhanced in the presence of GO nanosheets, regardless of crystallization temperature. The scale bar represents 100 μm for all images. GO0 stands for no graphene oxide [71].

Figure 15a demonstrates that in the absence of GO nanosheets formation of SCs is very limited and instead PLLA and PDLA spherules form. In the presence of GO nanosheets, however, many more stereocomplex crystals, SCs, form (Figure 15b). The microscope images in the bottom panel of Figure 15 clearly demonstrate this transformation from spherule-like crystal domains to diamond-like shaped crystals in the presence of GO at different crystallization temperatures.

## 5. Effect of Graphene on Mechanical Properties of PLA Polymers

Review of the literature on PLA-graphene nanocomposites in the form of free standing films produced either by solvent processing or by melt molding/extrusion indicates that there is still a significant lack of established recipe or nanocomposite fabrication protocol that could be used to enhance the elastic modulus or elongation at break values of PLA polymers using graphene-based materials. This is due to the fact that graphene comes as filler in the form of many stacked layers (known as GPs, GnPs etc.), which is influenced by the synthesis history. Different lateral size and thickness distribution exist in all these “commercially available” graphene products. Separately, graphene oxide or reduced graphene oxide introduces other parameters such as surface reactive sites and polarity effects. However, most recent reports highlight the necessity to functionalize graphene surfaces or use graphene oxide with graft chemistry [72] in order to compound in PLA polymers. For instance, Fu et al. [73] used an approach to modify the interfacial compatibility between graphene and polylactic acid (PLA) by manipulating the functionalization of graphene and introducing an epoxy-containing elastomer modifier. Curing between the functional groups of the modified graphene and the epoxy groups of the elastomer modifier resulted in controlled dispersion and distribution of graphene in the composite system and hence improved the interfacial adhesion between PLA and graphene. Effects of different graphene functionalization on morphology, viscoelasticity, and thermal properties of the resulting PLA nanocomposites were thoroughly examined. The resulting percolated structures were the origin of the improved properties of PLA/graphene nanocomposites. Storage modulus and complex viscosity measurements showed that due to crosslinking reaction between functionalized graphene oxide and the epoxy-modified rubber phase/PLA matrix, a three-dimensional percolated structure formed which was the origin of the improved viscoelasticity and thermal stability.

**Figure 16:**
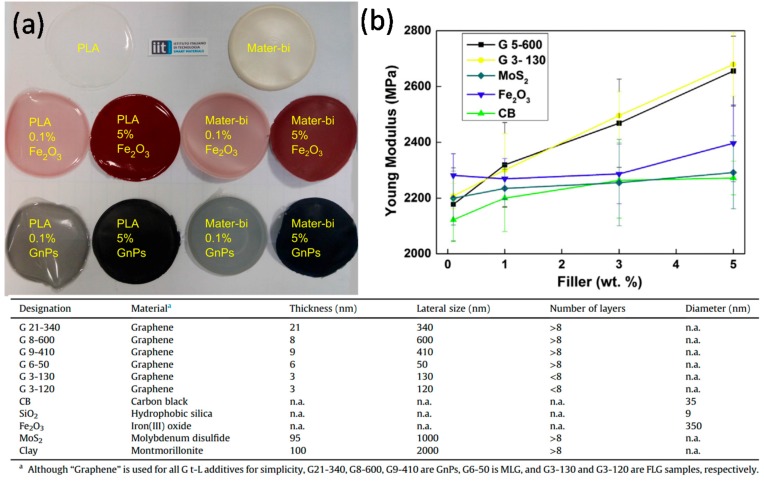
(**a**) Photograph of different solvent-cast composite films from PLA and Mater-Bi^®^ (Novamont, Italy) biopolymers. Transparent and whitish films featured on the top are two unfilled matrices; (**b**) Elastic modulus versus filler weight percent measurements for solvent-cast PLA polymer matrix composites; (**c**) The table lists dimensional characteristics of all the nanoscale fillers used [74].

Cataldi et al. [74] fabricated biocomposites by dispersing different graphene nanoplatelets (GnPs) as well as other non-graphitic 2D and 3D nanoscale particles (See table in Figure 16) in two representative bio-polyesters classified as soft (Mater-Bi, polycaprolactone based) and hard (PLA) matrices as shown in Figure 16a. Films were produced by solvent casting and hot-pressing. Hot-pressing was found to align the GnPs within the polymer matrices. The alignment had a more significant effect on the amorphous soft bio-polyester rather than PLA. In the case of solvent cast soft matrix biocomposites, GnPs did not induce any superior enhancements in elastic moduli compared to other non-graphitic 2D and 3D fillers.

In the case of PLA, however, both large multi- and few-layer GnPs caused up to 35% increase in elastic (Young’s) modulus compared to other model 2D or 3D fillers; see Figure 16b. As a result of hot-pressing, GnP flakes significantly increased the elastic modulus of the soft bio-polyester. In particular, large multi-layer GnPs induced up to 200% stiffness enhancement compared to few-layer GnPs and non-graphitic 2D fillers. Upon hot-pressing, about 15% stiffness enhancement levels were measured in the nanoparticle filled soft bio-polyester. Compared to solvent casting, hot-pressing of the PLA matrix nanocomposites did not yield better elastic modulus enhancements neither by large multi- nor few-layer GnPs. Stiffening levels remained the same.

**Figure 17:**
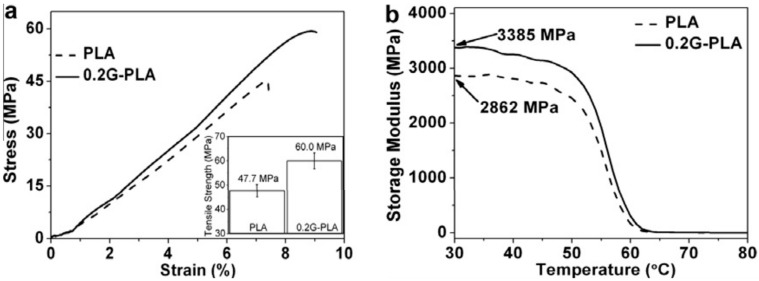
(**a**) Representative stress–strain curves; and (**b**) plots of storage modulus vs. temperature for PLA and 0.2GNS-PLA. The inset in (**a**) contains tensile strength values of PLA and 0.2GNS-PLA [75].

Cao et al. [75] produced solvent-free graphene nanosheets, which were in the form of chemically reduced graphite oxide nanosheets, using an environmentally friendly freeze drying (lyophilization) process. Compared to those produced through the vacuum filtration method, they claimed that the lyophilized graphene powders (GNS) were extremely light, loosely packed, and could be readily re-dispersed in organic solvents like N,N-dimethylformamide as individual sheets with the aid of sonication. This facilitated GNS powder incorporation into PLA through a solution-based processing method. In the resulting nanocomposites, GNSs were uniformly dispersed in the matrix and enhanced the mechanical and thermal properties of the host PLA polymer as shown in Figure 17a. They showed that GNS loading of 0.2 wt% (0.2G-PLA in Figure 17) was sufficient to enhance mechanical properties.

They reported that even for such a small percentage of GNS the reinforcing effect is rather significant: In other words, 0.2 wt% GNS incorporation leads to a 26% increase in tensile strength and a 18% raise in Young’s modulus. In their work [75], Young’s modulus was determined from DMA traces as storage modulus at 35 °C, at which PLA and 0.2G-PLA are both in the glassy state, as shown in Figure 17b.

**Figure 18:**
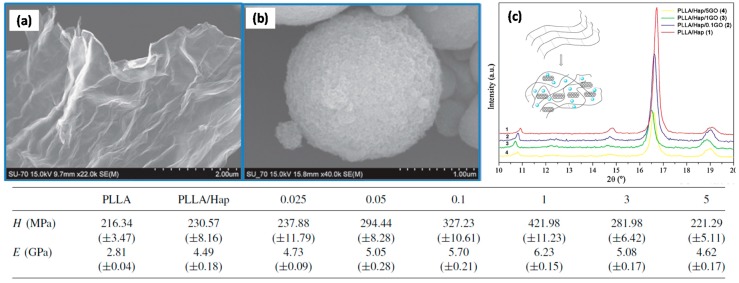
SEM images of (**a**) graphene oxide and (**b**) hydroxyapatide; (**c**) X-ray diffraction patterns of PLLA/Hap and PLLA/Hap with 0.1, 1, and 5% of GO. Inset: schematic representation of polymer chains with and without Hap and GO fillers. The table shows hardness and elastic modulus values of PLLA, PLLA/Hap, and PLLA/Hap with different GO percentages, measured by nano-indentation [76].

Marques et al. [76] reported a novel two-step method for the preparation of PLLA/hydroxyapatite (Hap)/GO nanocomposites with augmented mechanical properties when compared to PLLA/Hap and neat PLLA. The presence of graphene oxide (GO) had a positive effect on the dispersion of hydroxyapatite particles in the polymeric matrix with good homogenous final nanocomposite structure. PLLA nanocomposites prepared with 30% (w/w) of Hap and 1% (w/w) of GO showed the highest hardness and storage modulus values indicating an efficient load transfer between the fillers and the PLLA matrix. They postulated that these nanocomposites could be used in biomedical applications such as bone screws. They synthesized GO by the chemical exfoliation of graphite in aqueous media followed by ultrasonic processing, resulting in thin sheets of GO that readily formed stable colloidal suspensions in water. Their GO nanosheets were described as large flakes with lateral dimensions higher than 4 μm (see SEM image in Figure 18a).

Highly pure nanocrystals of Hap were used in the form of spray dried aggregated micron-size powders as can be observed in Figure 18b. To understand how GO influences the crystallinity of PLLA/Hap, they conducted X-ray diffraction (XRD) analysis. Figure 18c displays the diffraction patterns of PLLA/Hap and PLLA/Hap with 0.1, 1, and 5% GO nanocomposites. The diffraction from the crystalline regions that are present even in amorphous PLLA has several characteristic maxima, the two strongest are at 2*θ* of 17° and 19° corresponding to a typical PLLA pseudo-orthorhombic α structure. These peaks are also present in the XRD pattern of the nanocomposites (Figure 18c) but with lower intensity in the presence GO when compared with PLLA/Hap. They argued that this indicated a decreasing crystallinity of the polymeric phase of the nanocomposites with GO addition. Changes in the mechanical properties of the PLLA nanocomposites including different amounts of GO are summarized in the table in Figure 18. As seen, the overall mechanical properties of the resulting nanocomposites were enhanced and present a maximum improvement with the addition of 1% (w/w) of GO.

**Figure 19:**
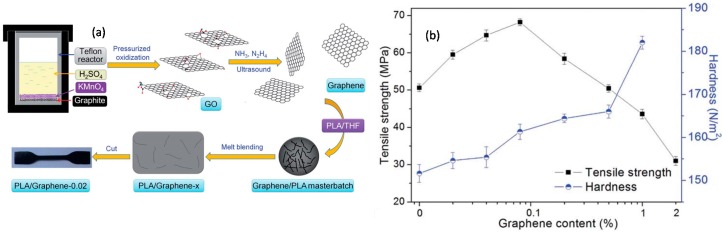
(**a**) Illustration for the preparation of graphene and PLA/graphene nanocomposites. The graphite is oxidized by pressurized oxidization and then reduced to single-atom-thick graphene by a multiplex reduction method. The graphene/PLA masterbatch (20% graphene) is prepared by solvent blending from PLA and graphene in THF media. The ‘‘x’’ in ‘‘PLA/Graphene-x’’ is the percentage of the graphene. The black sample is PLA/graphene-0.02 which contains 0.02% grapheme; (**b**) Tensile strength and hardness [77].

Bao et al. [77] produced graphene from graphite by pressurized oxidation and multiplex reduction. They executed this pressurized oxidation process with multiplex reduction based on ammonia and hydrazine and produced single-atom-thick graphene (0.4–0.6 nm thick; see Figure 19a). They then dispersed their graphene in PLA by melt blending. The graphene was found to be well dispersed and the obtained nanocomposites had markedly improved crystallinity, rate of crystallization, and mechanical properties. The properties were found to be dependent on the dispersion and loading content of graphene, showing a percolation threshold at 0.08 wt%. They argued that graphene had reinforced the PLA resin but after a critical concentration perturbed the interactions among the polymer chains, which led to somewhat reduced mechanical properties (see Figure 19b). Their tensile strength measurements indicated that tensile strength increases up to 0.08 wt% (a 35% increase) and then declines. The hardness of the PLA/graphene nanocomposites increases with the graphene content and the increase is about 30 N·mm^−2^ at 2 wt% graphene loading.

**Figure 20:**
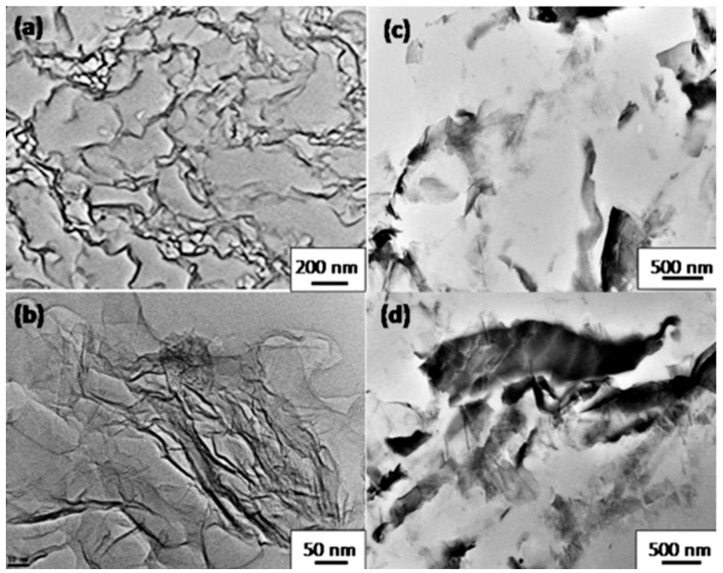
High resolution transmission electron microscope images of well-dispersed GnPs (**a**,**b**); and poorly-dispersed GnPs (**c**,**d**) in the PLA matrix. The graphene concentration in each figure was 0.56 vol% [78].

Sabzi et al. [78] prepared solvent cast PLA nanocomposite samples with two types of graphene nanoplatelets. They observed that one of the graphene samples was homogenously dispersed throughout the PLA matrix, mainly in single sheets (see Figure 20a,b), whereas the other was poorly delaminated and dispersed (Figure 20c,d). Well-dispersed GnPs had a surface area of ~600 m^2^/g, thickness of 1 nm with a diameter less than 10 microns; whereas poorly-dispersed GnPs had a surface area of ~140 m^2^/g, thickness of 7 nm with a diameter more than 25 microns. Dynamic rheological tests and theoretical modeling were conducted to characterize the structures and viscoelastic behavior of the graphene percolation networks.

Well-dispersed GnPs had a lower percolation threshold than poorly-dispersed GnPs because of the former’s larger aspect ratio, better dispersion, favorable surface structure, and chemistry. A well-dispersed GnP network with high elasticity and strength was established through high density direct inter-particle connections, whereas the poorly-dispersed GnP network was a polymer-bridged network which was formed at high GnP content and showed low elasticity (Figure 20).

Zhang et al. [79] prepared PLA/GO nanocomposites with enhanced thermomechanical and electrical properties by a solvent-casting method while blending commercial PLLA resin with GO-g-PDLA, which was synthesized via ring-opening polymerization in which GO acted as an initiator. The percolation concentration was shown to be 1.0 wt% and 0.5 wt% in PLLA matrix filled with pristine GO and GO-g-PDLA, respectively. Their in-situ FTIR showed that the SCs grew from the GO surface and stereocomplex crystallite networks were formed with the enhanced GO network in PLLA/GO-g-PDLA composites, while only stereocomplex crystallite networks consisted of spherocrystal (spherical crystalline mass) and PLA chains in PLLA/PDLA composites. Remarkable improvement in crystallinity of stereocomplex nanocomposites was observed because of the heterogeneous nucleating effect of GO-g-PDLA. They demonstrated that the high-temperature stereocomplex crystallite platform appeared when the content of PDLA and GO-g-PDLA reached 15 and 10 wt%, respectively. The formation of stereocomplex crystallite on the surface of GO increased the strength of stereocomplex crystallite networks and significantly improved the storage modulus at 195 °C for PLLA/GO-g-PDLA mixtures at various compositions.

**Figure 21:**
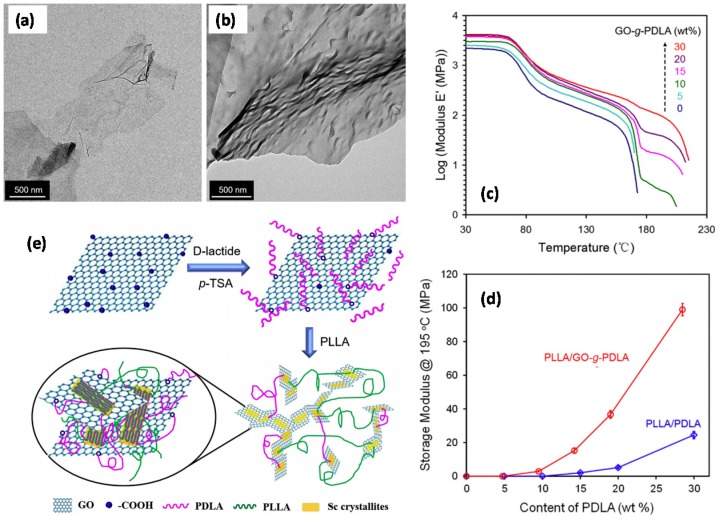
TEM images of (**a**) GO and (**b**) purified GO-*g*-PDLA. Temperature dependency of storage modulus (*E*’) for PLLA mixed with various concentrations of (**c**) GO-*g*-PDLA. (**d**) Variation in storage modulus at 195 °C as a function of the PDLA content for PLLA/PDLA and PLLA/GO-*g*-PDLA mixtures, respectively. (**e**) Schematic showing the synthesis of GO-g-PDLA by the direct melt-polycondensation approach and the formation of GO enhanced stereocomplex (Sc) network. p-TSA stands for p-toluenesulfonic acid [79].

Figure 21a displays GO flake which is about 1 micron in the size later used by Zhang et al. [79], and in Figure 21b PDLA a grafted GO crystal is displayed. As shown in Figure 21c, storage modulus (*E*’) at 30 °C increased from 2.2 GPa to 4.1 GPa with the addition of 20 wt% GO-g-PDLA (containing 1 wt% GO). The large mechanical enhancement was ascribed to the reinforcement of GO on the PLLA–PDLA stereocomplex networks. Similarly, this effect is more significant at 195 °C, particularly after 10 wt% PDLA inclusion. They argued that existence of GO can be regarded as the landscape of the ordering of SC helices surrounding the PLA/GO interface. At last, the stereocomplex crystallite, SC, could begin to grow from the GO surface with the hydrogen bonding inter-chain interactions between PLLA and PDLA and finally an SC network would form with the link of PLA chains and enhancement of GO as schematically shown in Figure 21e.

**Figure 22:**
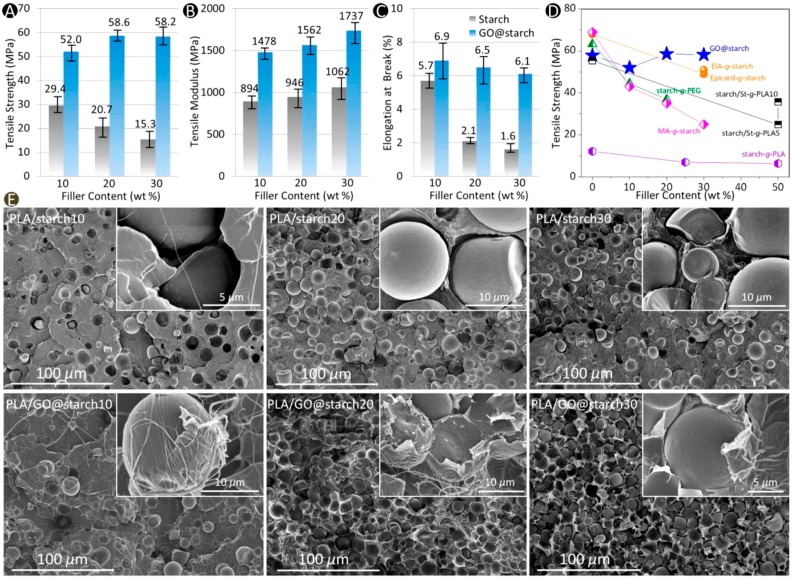
Mechanical properties of composite films. (**A**) Tensile strength; (**B**) tensile modulus; and (**C**) elongation at break demonstrating the superior tensile properties of PLA/GO@starch in comparison with PLA/starch; (**D**) Comparison of tensile strength for modified PLA/starch composites using the present method and various published covalent grafting techniques, including epoxidized itaconic acid (EIA)-*g*-starch, epoxidized cardanol (Epicard)-*g*-starch, combined use of starch and starch-g-PLA (5 and 10 wt%), starch-g-poly(ethylene glycol) (PEG), maleic anhydride (MA)-g-starch and starch-g-PLA. See references in [80] related to each grafting method; (**E**) SEM images of fracture surfaces after tensile failure suggesting strong interfacial bonding and even formation of numerous ligaments in PLA/GO@starch films [80].

Xu et al. [80] demonstrated a very interesting way to use GO in enhancing thermomechanical properties of PLA. In their study, a thin layer of GO nanosheets was immobilized onto starch granule surfaces by hydrogen bonding to create active GO@starch surfaces. Once incorporated into the PLA matrix, GO@starch allowed formation of nanointerfaces between PLA matrix and starch particles, without migration of GO into the PLA matrix. Although the GO layer was ultrathin and GO was present at ultralow concentration (up to 0.03 wt%), these nanointerfaces significantly enhanced PLA–starch interfacial interactions with uniform dispersion of GO@starch in PLA matrix. Driven by the high surface activity of high-aspect-ratio nanosheets, 50% enrichment in crystallinity was demonstrated for PLA/GO@starch films; clearly surpassing that of PLA/starch composites (below 15%). They demonstrated that the combination of high tensile strength (58.2 MPa), improved elongation (6.1%) (see Figure 22A–D) and superior low gas barrier properties, outperforming PLA/starch30 with an increase of ~280% in both strength and elongation, and a drastic decline in 81.6% in oxygen gas barrier. Microscale morphologies of PLA/starch and PLA/GO@starch composites are presented in Figure 22E. PLA/GO@starch interfaces demonstrate much better bonding via fibrillar ligaments, which did not form in PLA/starch composites.

## 6. Scaffolds Based on PLA-Graphene Nanocomposites: Porous Networks, Nanofibers, and Foams

In biomedicine, polymeric muscle or bone scaffolds must have an appropriate porosity range with an interconnected and open porosity, and have a biodegradable rate and mechanical properties that match the injured tissue. PLA polymers have been extensively studied for this purpose [81]. As expected, graphene or graphene derivatives have also been incorporated in PLA based scaffolds to tune and enhance various thermomechanical attributes suitable for biomedical engineering.

**Figure 23:**
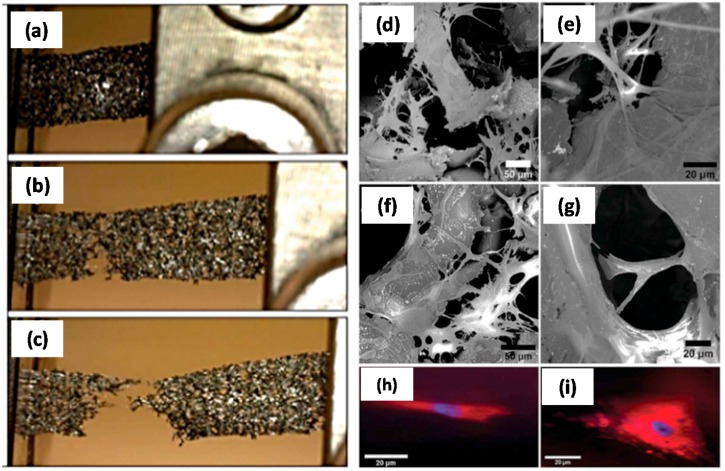
(**a**) GrF-PLC sample in tensile test setup; (**b**) GrF-PLC sample in tension showing signs of necking; (**c**) GrF-PLC after tensile failure, load prior to failure can be seen to be borne by a few high strength strands. (**d**,**e**) SEM images of graphene foam scaffold with human mesenchymal stem cells (hMSCs); (**f**,**g**) SEM images of graphene foam-PLC hybrid scaffold with human mesenchymal stem cells (hMSCs); (**h**) Fluorescence microscopy image of stem cells on graphene foam, cells appears severely stretched and thinned; (**i**) Fluorescence microscopy images of stem cells on graphene foam-PLC scaffold [82].

Nieto et al. [82] demonstrated high strength biocompatible scaffolds synthesized by dipping graphene foam (GrF) into a liquid solution containing PLA–poly-*ε*-caprolactone (PCL) copolymer, designated as PLC. Exceptional wettability of PLC on graphene foam resulted in PLC forming a thin uniform coating over the graphene foam. This effective wettability allowed the PLC to fill voids and defects that had been present in the graphene foam. The healing of defects in conjunction with the formation of PLC bridges caused enhanced strength and ductility in the GrF-PLC scaffold. They conducted nano-indentation and macroscale tensile tests and demonstrated that significant increase in strength in both compression and tension at multiple scales occurred, as shown in Figure 23 (see the left panel). Further, the effectiveness of PLC bridges bearing load was observed directly via in situ tensile testing in an SEM. Cellular studies conducted on both graphene foam and GrF-PLC scaffolds demonstrate the ability of human mesenchymal stem cells (hMSCs) to survive and proliferate throughout both scaffolds. The higher strength of the GrF-PLC scaffold enables the hMSCs to grow normally without undergoing large deformations. The pure graphene foam does not have sufficient strength to withstand cell-induced strains and leads to hMSCs growing with highly elongated morphology (Figure 23e), whereas in Figure 23f cells grown on GrF-PLC scaffold have larger and more rounded morphology indicating normal growth.

**Figure 24:**
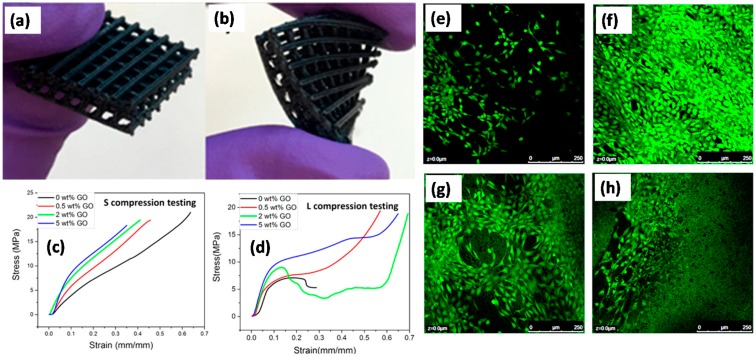
(**a**) 3D printed microlattice (5 wt% of GO); (**b**) 3D printed microlattice under bending (5 wt% of GO); (**c**) S Compression testing curves of samples of different GO loadings; (**d**) L Compression testing curves of samples of different GO loadings. 96 h cell culture results of NIH3T3 cells on 3D printed TPU/PLA with different GO loadings: (**e**) 0 wt% GO; (**f**) 0.5 wt% GO; (**g**) 2 wt% GO; (**h**) 5 wt% GO. Green color indicates live cells, whereas red color indicates dead cell [83].

Chen et al. [83] produced 3D printed elastic thermoplastic polyurethane/PLA/GO or TPU/PLA/GO nanocomposites by using a solvent mixing process as well as a fused deposition modeling technique, as displayed in Figure 24a,b. Nanocomposites were printed into complex shapes with high resolution. Addition of GO significantly enhanced the mechanical properties of the polymer matrix, 167% in compression modulus, and 75.5% in tensile modulus (see Figure 24c,d). Printing orientation led to different mechanical responses due to the weak adhesion strength between layers during 3D printing. To investigate the effect of the addition of GO on the mechanical property of the 3D printed parts as well as the effect of printing orientation, compression testing and tensile testing were performed. For compression testing, all samples were printed into a cuboid shape specimen. To investigate the effect of printing orientation on mechanical response, each type of sample (including TPU/PLA blend without GO, with 0.5, 2, and 5 wt% of GO) was prepared in two different printing orientations: standing specimen and lying specimen. For the standing specimen, the printing orientation is the same as the height direction while for the lying specimen; the printing orientation is the same as the width (length) direction. Both types of specimens were performed under compression testing in such a way that the height direction is parallel to the compression direction. Therefore, two kinds of compression testing were performed on each sample: “S compression testing”, where the standing specimen is used in the testing, and “L compression tests”, where the lying specimen is used in the testing. The data are shown in Figure 24d,e. Compression modulus in both L and S compression constantly increased with the loading of GO. Modulus increased 56% in L compression and 167% in S compression at 5 wt% GO. They argued that addition of GO had significantly improved the compression strength of TPU/PLA matrix.

Thermal stability was also shown to be improved, with 90 °C increase in degradation temperature as well as the new formation of better crystalline structures. They also conducted cell culturing experiments and results revealed excellent cell viability (Figure 24e–h) of 3D printed scaffolds, indicating limited addition of GO has no obvious toxicity to cell growth, and small amount of GO is beneficial for cell proliferation. They claimed that these 3D printed scaffolds can be good potential candidates for tissue engineering applications.

Chen et al. [83] further printed thin sheets of TPU/PLA blends containing 0.5, 2, and 5 wt% GO to support cell adhesion, growth, and proliferation. These 3D printed TPU/PLA GO monolayers were evaluated as seeding scaffolds using NIH3T3 mouse embryonic fibroblast cells with a LIVE/DEAD viability/cytotoxicity assay. The results of the LIVE/DEAD assay indicated that at all scaffolds, supported cell growth as only live cells was detected; no dead cells were detected in any scaffold (Figure 24e–h). Additionally, the NIH3T3 cells spread well and proliferated on all scaffolds. Although all scaffolds supported cell growth and proliferation, the TPU/PLA with 0.5% GO (Figure 23f) showed the highest density of cells across all loadings of GO and even when compared to the TPU/PLA control. They attributed this to the configuration of GO in the TPU/PLA scaffold. If the loading level of GO is too high, the GO may not “lay” flat in the polymer matrix and thus the enhancement in cellular growth may not advance. Taken together, they claimed that all scaffolds were nontoxic toward embryonic cells with 0.5% GO providing the greatest enhancement in cellular growth and proliferation as indicated by the LIVE/DEAD assay.

**Figure 25:**
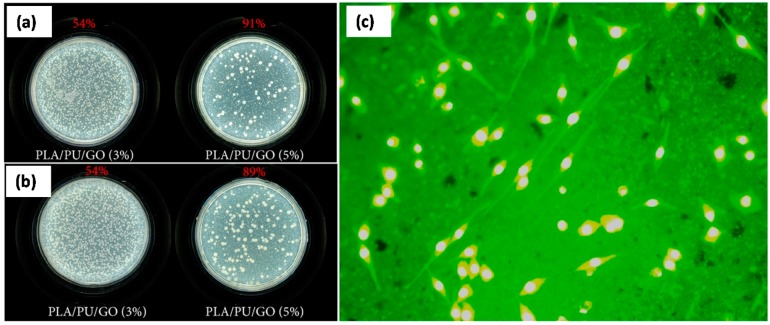
Photographs of (**a**) *S. aureus* and (**b**) *E. coli* grown on PLA/PU/GO (3%) and PLA/PU/GO (5%) for 4 h, respectively; (**c**) Fluorescence microscopy image of MC3T3-E1 cells grown on the electrospun PLA/PU/GO (5%) nanofibers for 48 h at 37 °C. The magnification is 100× [84].

An et al. [84] produced porous films and nanofiber nanocomposites from PLA/Polyurethane (PU) blends containing small concentrations of GO by simple liquid-phase mixing followed by casting. The as-prepared ternary PLA/PU/GO composite films exhibited good antibacterial activity against the Gram-positive *Staphylococcus aureus* and the Gram-negative *Escherichia coli*. They attributed this to the inherent antibacterial property of GO sheets with high specific surface area (see Figure 25a). Addition of GO inhibited attachment and proliferation of microbes on the film surfaces, resulting in remarkably improved antibacterial activity compared to PLA/PU composite film. The inhibition efficiency was found to be proportional to the amount of GO (Figure 25a). Furthermore, they fabricated PLA/PU/GO composite fibrous surfaces using electrospinning with good biocompatibility. Incorporation of GO did not destroy normal cell’s proliferation and differentiation as shown in Figure 25b,c. Even though they did not present any thermomechanical properties of the composites they concluded that PLA/PU/GO composites with good antibacterial activity and biocompatibility could render them attractive for clinical applications such as tissue engineering.

**Figure 26:**
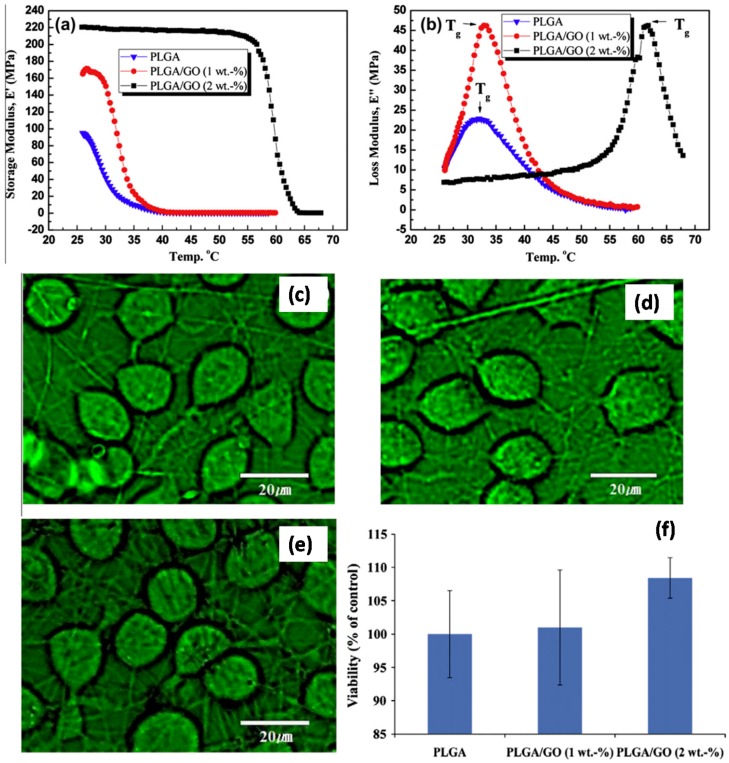
Dynamic mechanical analyzer curves as a function of measurement temperature. (**a**) Storage modulus and (**b**) loss modulus. For PLGA, PLGA/GO (1 wt%), and PLGA/GO (2 wt%) nanofibers. Optical microscope images of PC 12 cells on (**c**) PLGA nanofibers; (**d**) PLGA/GO (1 wt%) nanocomposites; (**e**) PLGA/GO (2 wt%) nanocomposites; and (**f**) cell proliferation and viability data obtained by WST-1 assay of the cells cultured for 2 days (*n* = 12, *p* <0.05) [85].

Yoon et al. [85] constructed nanocomposite nanofibers of poly(d,l-lactic-co-glycolic acid) (PLGA) embedded with GO nanosheets by electrospinning, and investigated their physiochemical properties including morphological, surface-chemical, mechanical, and thermomechanical properties. They showed a 2-fold increase in tensile modulus as compared to that of pristine PLGA nanofibers. Storage modulus and glass transition temperatures of the PLGA/GO (2 wt%) nanocomposites were much higher than those of PLGA nanofibers and PLGA/GO (1 wt%) nanocomposite nanofibers, as shown in Figure 26a,b.

Interestingly they claimed that the incorporation of GO nanosheets according to concentration led to effective enhancement of their mechanical and thermomechanical properties compared to those of pristine PLGA nanofibers. This enhancement was attributed to stronger interfacial interactions caused by stronger chemical bonding between the PLGA and GO nanosheets and the axial alignment of the GO nanosheets embedded nanocomposite nanofibers. They also showed that nanocomposite nanofibers were more hydrophilic than PLGA nanofibers. In order to investigate the effects of GO nanosheets on nanocomposite biocompatibility, PC 12 neuronal cells were cultured for two days on various nanofibrous scaffolds, and the proliferation and viability of the cells were measured. Biocompatibility tests (Figure 26c–f) showed that the enhanced surface chemical properties of the PLGA/GO (2 wt%) nanocomposites effectively improved neuronal cell proliferation and viability (Figure 26f), indicating enhanced biocompatibility of PLGA nanocomposite nanofibers by the addition of 2-D GO nanofillers.

Zhang et al. [86] surface grafted GO with polyethylene glycol (PEG) to improve its interfacial adhesion with PLA. Both GO and GO-*g*-PEG were incorporated in PLA polymer as reinforcing fillers to fabricate nanofibrous scaffolds of PLA, PLA/GO, and PLA/GO-*g*-PEG, which were prepared by means of electrospinning. They measured decreases in complex viscosity for PLA/GO-*g*-PEG electrospinning suspensions and attributed these observations to the plasticizing effect of the grafted PEG molecules on the surface of GO.

**Figure 27:**
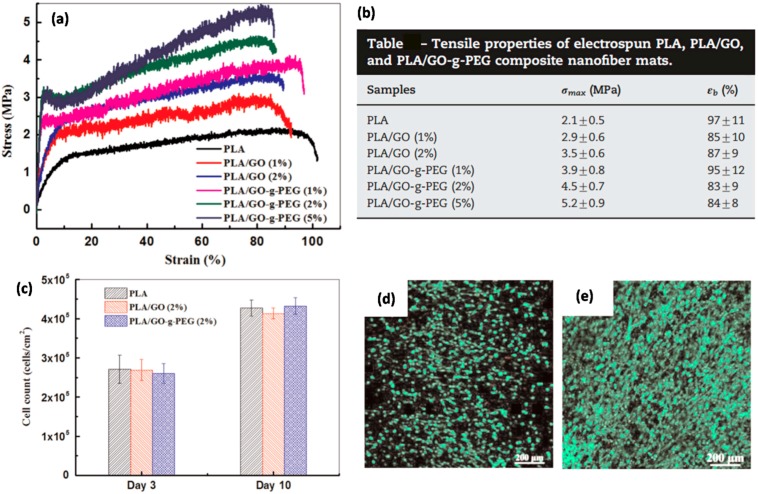
(**a**) Typical stress–strain curves of electrospun PLA, PLA/GO, and PLA/GO-*g*-PEG composite nanofiber mats; (**b**) Table demonstrating elastic modulus (σ_max_) and elongation at break values (ε_b_); (**c**) Proliferation cell counts of NIH 3T3 cells on scaffolds after 3 and 10 days of culture. Fluorescence micrographs of stained cells showing live (green) and dead (red) cells on PLA/GO-g-PEG (2%) scaffolds after (**d**) 3 and (**e**) 10 days of culture [86].

This indicated good interfacial adhesion between GO-g-PEG and PLA and accounted for the decrease in the average fiber diameter from PLA to PLA/GO-g-PEG. Based on the TGA results, GO-*g*-PEG had a greater influence than GO in improving the thermal stability of PLA. Tensile tests showed that the addition of GO and GO-*g*-PEG in PLA achieved significantly improved tensile stress, as shown in Figure 27a,b. However, GO-*g*-PEG was more effective in reinforcing PLA than GO due to the grafted PEG chains, which improved the filler dispersion as well as the adhesion between GO and PLA. Moreover, PLA/GO and PLA/GO-*g*-PEG composite nanofibrous scaffolds were found to be non-toxic to NIH 3T3 cells and were capable of supporting cell attachment and growth (see Figure 27c,d). They claimed that their PLA/GO-*g*-PEG scaffolds with enhanced mechanical strength and good cytocompatibility could be promising for applications in tissue engineering. This study is unique in the sense that it demonstrated both improved thermomechanical properties due to GO as well as promising cytocompatibility.

Pal et al. [87] formulated PLA scaffolds loaded with different percentage of cellulose nanocrystals (CNC) and reduced GO, rGO, as reinforcing nanofillers by a solution casting method. The modification and effects of the incorporated CNC and rGO on the morphology, crystallinity, thermal, mechanical, and bio-medical properties were analyzed. Scaffolds with the percentage of CNC (1%) and rGO (0.5%) were displayed improved biocompatibility, thermal and mechanical properties. For instance, an increase of 23.35% in tensile strength and 28.71% in Young’s Modulus, with respect to neat PLA was observed in the scaffolds along with increase in thermal degradation threshold temperatures. Cytocompatibility tests proved that the PLA/CNC/rGO scaffolds were not toxic to NIH-3T3 cells. This was also supported by the morphological examination of treated cells. Moreover, the scaffolds were shown to have antibacterial potential against Gram positive *Staphylococcus aureus* (*S. aureus*) and Gram negative bacteria *Escherichia coli* (*E. coli*) as well. Although promising results were reported by Pal et al. [87], further in vivo tests along with biodegradation dynamics need to be studied in order to ascertain applicability for biomedical uses.

Luo et al. [88] fabricated GO-incorporated PLGA nanofibrous mats via an electrospinning approach and explored the materials use as scaffold materials for tissue engineering. GO was readily electrospun into PLGA nanofibers without changing the 3D porous structure. The impact of the GO incorporation on the mechanical strength of the PLGA nanofibrous mat was also probed. They concluded that all the materials possess excellent mechanical properties. But the breaking strength and Young’s modulus decreased after the addition of 1% GO. This was attributed to its 2D topological plane structure, GO might tend to be vertical to the fibers. They further stated that when the fibers are under stress, GO cannot transfer the part force, leading to the decrease of breaking strength. The GO-doped PLGA nanofibrous mats could sustain the extracellular matrix (ECM) biomimetic microenvironment for human mesenchymal stem cells (hMSCs) adhesion and proliferation. They claimed that these GO-incorporated PLGA nanofibers could serve as novel tissue engineering scaffolds with good biocompatibility. Furthermore, they demonstrated that the GO-doped PLGA substrate induced expression of osteogenic marker genes such as ALP, Col I, and Ocn. Meanwhile, it promoted ALP activity and osteocalcin secretion. The PLGA nanofibers incorporated with GO not only promoted the attachment and proliferation of hMSCs but also enhance the hMSCs differentiation toward osteoblast, which is important for biomedical applications requiring the biomimetic of extracellular matrix (ECM).

Kuang et al. [89] investigated the influence of graphene oxide (GO) on the unidirectional foaming of PLA using supercritical CO_2_ as blowing agent. PLA/GO nanocomposite foams with oriented and highly elongated cell structure were prepared for the first time in this study. They prepared the nanocomposites by a solution mixing method at various GO contents. The thermal and rheological properties, and CO_2_ absorption ability of the composites, were investigated and compared with neat PLA. It was found that the incorporation of GO improved the crystallinity of the PLA matrix. The addition of GO enhanced the storage modulus, loss modulus, and complex viscosity of the PLA matrix as well, and the improvements increased with increase of GO content. The unidirectional microcellular foaming revealed that the PLA/GO nanocomposites had the ability to form highly elongated cell structure. Compared to neat PLA, the relatively high GO content (i.e., >0.6 wt%) in PLA/GO nanocomposites showed significantly higher expansion ratio, average foam cell size, and cell diameter ratio in the long axis and minor axis. These behaviors were caused by the enhanced CO_2_ absorption rate, PLA/GO matrix viscosity, and the unidirectional foaming process. They did not demonstrate biocompatibility or discuss any potential biomedical applications although their cellular architectures were claimed to be very suitable as 3D biomedical scaffolds.

## 7. Conclusions and Outlook

Although PLA polymer is regarded as one of the most suitable biopolymers for biomedical applications with many successful in vitro and in vivo demonstrations to date, it is now understood that homo-composites of PLA polymers are not only thermally and mechanically superior to pure PLA but also perform better as drug release platforms [90]. In other words, by homo-composite blending PLLA and PDLA or synthesis of stereo block PLA, a stereocomplex (SC), is formed. PLA SC polymers have higher melting temperatures (or heat resistance), mechanical performance, and hydrolysis-resistance compared to those of neat PLLA and PDLA. As such, they have been extensively studied in terms of biomedical and pharmaceutical applications as well as commodity, industrial, and environmental applications [32,91]. Stereocomplexation stabilizes and strengthens PLA-based hydrogel or nanoparticles for biomedical applications, as well. Stereocomplexation increases the barrier property of PLA-based materials and thereby prolongs drug release from PLA based materials. Table 1 presents a summary of the work reviewed herein. The table indicates that more work should be devoted to SC blends, and the majority of the works have been conducted by solvent instead of melt processing and somewhat lack proper thermomechanical characterization.

On the other hand, clearly, graphene has the ability to modulate crystallinity and the final thermomechanical properties of PLA polymers including SCs. However, the effectiveness lies in the proper surface functionalization of graphene of GO, a priori. As such, compatibilization of graphene or graphene derivatives with PLA SCs can yet produce significant improvements in the thermomechnical properties of SCs [92]. To achieve this PLA-graphene interface engineering is needed in order to be able to enhance the thermomechanical properties of PLA polymers by graphene incorporation [93]. In addition, more systematic works need to be conducted in order to establish the effect of the type of graphene nanosheets in terms of lateral size and number of layers on the thermomechanical properties of PLA polymers and at the same time to maintain acceptable biocompatibility levels for potential clinical applications [94].

An outstanding biomedical issue that still needs to be addressed is the fact that various chemical pathways and agents used or proposed for surface functionalization or grafting graphene or graphene oxide nanosheets so that they can be inserted into PLA matrices must not yield cytotoxicity. Furthermore, their fate after passing through the cell membranes should be carefully evaluated. This issue is still open; however, encouraging new results are being published [95,96]. Similarly, the biological applications of graphene derivatives have significant potential because many attempts have shown promising results regarding bio-functionalization and standardization of graphene derivatives by fractionation based on size, number of layers, and chemical functionalities [33,96]. Therefore, it is not surprising that PLA/graphene material systems offer many unique advantages and chances to discover new fascinating properties and potential applications.

So far many published works on PLA/graphene composites exclusively focus either on the thermomechanical properties or on cell compatibility and behavior. Hence, none of them can be explicitly singled out as an ideal candidate for clinical applications. In fact, both properties are interrelated as was shown by some recent studies on the effect of PLA crystallinity on cell proliferation. Therefore, it is suggested that future studies on PLA-graphene systems should focus on surface functionalization of graphene and graphene oxide with biomacromolecules and incorporation of these modified graphene nanoflakes into PLA polymers, particularly into PLA stereocomplexes. The characterization should clearly demonstrate changes in crystallinity, thermomechanical properties due to functionalized graphene addition, and how this translates into cell proliferation and cytocompatibility compared to simple PLA polymer/graphene systems.

Since PLA polymers can be processed with ease in many different ways such as solution, spinning, extrusion, and injection, they offer unique possibilities to produce graphene–PLA nanocomposites with higher-order 3D architectures such as sponge-like macroporous scaffolds, nanofiber mats or hollow micrometer-sized spheres, which are ideal platforms for surgical reconstruction and drug delivery [97]. Although graphene and GO are able to accelerate the growth, differentiation, and proliferation of stem cells, and therefore hold great promise in tissue engineering, regenerative medicine, and other biomedical fields, still many safety questions exist (i.e., cellular uptake mechanisms) hence incorporation of graphene and/or GO in 3D PLA architectures can minimize and even eliminate these concerns.

The future seems to be the combination of PLA stereocomplexes with graphene and GO rather than PLA as PLA stereocomplexes are not only thermomechanically superior but also more flexible in terms of interfacial engineering, opening up many promising additional applications in delivery of drugs, genes, proteins, growth factors, and other biomolecules for cancer and other disease therapies. As seen, advances in PLA/graphene material systems are growing rapidly and this trend, the author believes, will continue and even speed up in the years to come.

## Figures and Tables

**Table 1:**
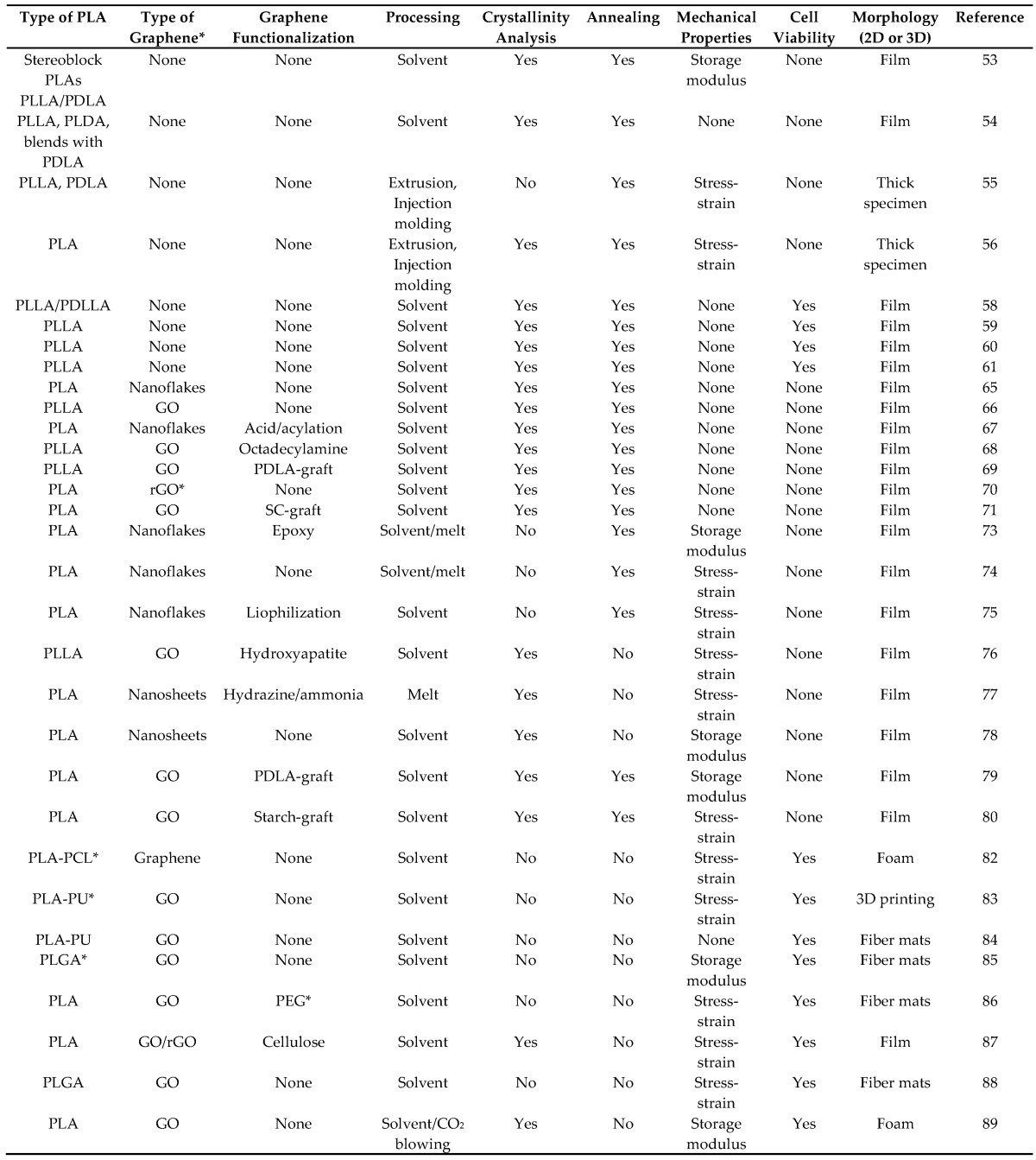
Summary of literature reviewed. The table lists the type of PLA polymer and graphene used as well as the processing conditions and characterization methods employed.

* rGO: reduced graphene oxide; PCL: Polycaprolactone; PU: Polyurethane; PEG: Polyethylene glycol; PLGA: Poly(lactic-*co*-glycolic acid).
